# Which factors affect the live birth outcome of the first single euploid frozen-thawed blastocyst transfer in couples with balanced chromosomal translocations?

**DOI:** 10.3389/fendo.2024.1378635

**Published:** 2024-04-26

**Authors:** Ruixiao Zhang, Yahui Hu, Chenchen Cui, Cuilian Zhang

**Affiliations:** ^1^Reproductive Medicine Center, Henan Provincial People’s Hospital, Zhengzhou, China; ^2^Reproductive Medicine Center, Zhengzhou University People’s Hospital, Zhengzhou, China; ^3^Reproductive Medicine Center, Henan University People’s Hospital, Zhengzhou, China

**Keywords:** balanced chromosomal translocations, preimplantation genetic testing for structural rearrangements (PGT-SR), euploid, frozen-thawed blastocyst transfer, live birth

## Abstract

**Objective:**

The objective of this study is to investigate the factors that influence the live birth rate (LBR) of the first single euploid frozen-thawed blastocyst transfer (FBT) cycles after preimplantation genetic testing for structural rearrangements (PGT-SR) in couples with balanced chromosomal translocations (BCT).

**Design:**

Single center, retrospective and observational study.

**Methods:**

A total of 336 PGT-SR and the first single euploid FBT cycles between July 2016 and December 2022 were included in this study. The patients were divided into two groups according to the live birth outcomes. The parameters of the study population, controlled ovarian stimulation cycles, and FBT cycles were analyzed. Multivariable binary logistic regression was performed to find the factors that affected the LBR.

**Results:**

The percentage of blastocysts at developmental stage Day 5 compared to Day 6 (51.8% vs. 30.8%; P<0.001) and with morphology ≥BB compared to <BB (49.7% vs. 32.2%; P=0.001) was significantly different between the group that resulted in live births (n=193) and the group that did not (n=143). The results of the multivariable binary logistic regression analysis indicated that the developmental stage (adjusted OR: 2.068, 95%CI 1.291-3.313; P=0.003) and morphology (adjusted OR: 1.697, 95%CI 1.039-2.773; P=0.035) of the blastocyst were significantly correlated with live birth. Patients with blastocysts that reached the developmental stage at Day 5 and had a morphology of ≥BB had a higher likelihood of having a live birth.

**Conclusion:**

The developmental stage and morphology of blastocyst affect the live birth outcome of the first single euploid FBT in BCT carriers undergoing PGT-SR.

## Introduction

There are two main types of structural chromosomal rearrangements: reciprocal translocations and Robertsonian translocations. Reciprocal translocation have an incidence of 1/700 in healthy individuals, while Robertsonian translocations have an incidence of 1/1000 (1). These are also known as balanced chromosomal translocations (BCT) because they do not involve significant loss of chromosomal material. Most carriers of BCT are phenotypically normal and may not even be aware of their carrier status until they experience infertility, miscarriage, or the birth of a child with congenital anomalies due to the chromosomally abnormal embryos (2). Preimplantation genetic testing for structural rearrangements (PGT-SR) which selects euploid blastocysts, can increase the chance of having a healthy live birth, reduce the rate of pregnancy loss, and shorten time to pregnancy (3), although the value of PGT-SR is still under debate (4, 5).

The reported live birth rate (LBR) per single euploid frozen-thawed blastocyst transfer (FBT) is 50-60% (6, 7). There is still room for improvement. Several studies have focused on the factors that influence the LBR of euploid FBT afterpreimplantation genetic testing for aneuploidies (PGT-A), as reviewed by Cimadomo et al. (8). The study found that blastocysts of poor quality, blastocysts that were developed on day 6-7, blastocyst that were frozen twice(though biopsied only once) maternal age of 38 years or older, a body mass index (BMI) of 30kg/m^2^ or higher, and a history of repeated implantation failure (RIF) were significantly associated with low LBR (8). PGT-A is recommended for women with advanced maternal age (AMA), recurrent miscarriage (RM) with normal parental karyotypes, and RIF (3). In PGT-A cycles, 40% of biopsied blastocysts were euploid (9), and all aneuploids were *de novo* sporadic. Apart from the embryo chromosomal abnormalities, the etiologies of RM and RIF are complex, such as thrombophilia, immunological factors, endocrine dysfunction, uterine endometrium, and lifestyle (10, 11). In BCT carriers, only 26% of the diagnosed blastocysts were found to be euploid (12, 13). This is due to the fact that BCT can result in gametes with a high risk of partial duplications, deletions, and whole chromosome aneuploidies. Additionally, there may be some *de novo* abnormalities of chromosomes that are not related to the rearrangement (14). Theoretically, embryonic chromosomal factors account for a large proportion of adverse pregnancies in BCT carriers compared to RM and RIF populations. However, carriers of BCT may experience frustration when a certain number of euploid FBT do not result in a live birth, but instead in failure of implantation or miscarriage. Few studies have evaluated the factors that influence LBR after a single euploid FBT in patients with BCT.

The objective of this study was to investigate the factors that affect the LBR of single euploid FBT in BCT carriers. The findings may offer supplementary information for the genetic counseling and clinical strategies for this specific group of couples who experience anxiety and stress.

## Materials and methods

### Study design and population

This was a single-center retrospective study of patients with BCT who underwent PGT-SR and had their first single euploid FBT at the Reproductive Medicine Center of Henan Provincial People’s Hospital affiliated with Zhengzhou University between July 2016 and December 2022.

The study’s inclusion criteria required that either the female or male in the couple was a carrier of BCT, that there were euploid blastocysts available for transfer after PGT-SR, and that the patient had undergone the first single euploid FBT cycle. To avoid confounding by patient-specific factors, only one cycle per patient was included in the study.

**Figure 1:**
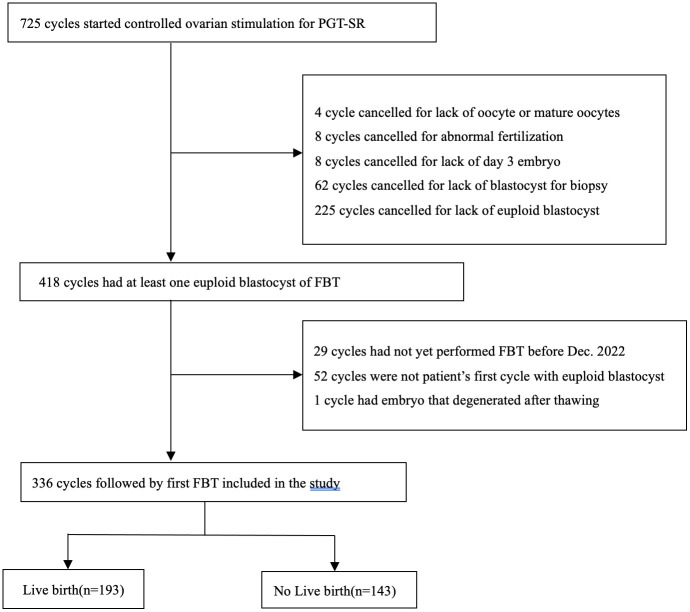
Flow chart of cycles’ selection.

The exclusion criteria for this study were as follows: (1) PGT-SR was conducted for reasons other than parental balanced translocation; (2) embryos were unavailable for biopsy or there was a lack of euploid blastocysts for transfer; (3) it was not the first cycle in which a euploid blastocyst was transferred; and (4) FBT was not performed before December 2022. All data were extracted from the medical database. In the end, the study included 336 cycles, as depicted in **Figure 1**.

All couples underwent genetic counseling and were informed of the advantages and limitations of PGT-SR procedures. They signed the informed consent for PGT-SR therapy.

### Treatment protocols

The procedures for controlled ovarian stimulation, oocyte retrieval, embryo transfer, and luteal-phase support were carried out according to our standard protocols, as previously described (15, 16). The clinical team utilized controlled ovarian stimulation protocols and gonadotropin doses tailored to each individual patient’s characteristics, such as age, BMI, ovarian reserve function, follicle conditions, hormone levels, differences in drugs, prior response to gonadotropins, financial situation, and schedule. The protocols used included GnRH agonist, flexible GnRH antagonist, and progestin-primed ovarian stimulation (PPOS). Final oocyte maturation was induced using a human chorionic gonadotropin (hCG) trigger (Lizhu Pharmaceutical Trading, China) and/or GnRH agonist (Triptorelin Acetate, Ferring, Switzerland) with the dose determined by the number of dominant follicles and peak estradiol level. Oocyte retrieval was then performed 34-36 hours later under transvaginal ultrasound guidance.

In all cycles, fertilization was achieved using intracytoplasmic sperm injection (ICSI). Embryos were cultured until the blastocyst stage on Day 5-6 and those with a grade ≥3BC according to Gardner criteria (17) were selected for trophectoderm biopsy, which was performed by experienced embryologists. Embryos with a grade of 3-6 AA/AB/BA/BB were considered good quality, while others were classified as poor quality. Whole genome DNA from approximately 5 cells per blastocyst was amplified and tested using next-generation sequencing (NGS) technology.

After the biopsy, the blastocysts were vitrified and stored in liquid nitrogen. During the frozen-thawed cycle, only one euploid blastocyst was transferred. The endometrial preparation protocol was determined based on the patient’s condition, either a natural cycle or a hormone replacement therapy (HRT). Luteal-phase support was initiated either on the day of ovulation or when the endometrial thickness was ≥ 8mm after taking oral estrogen (4 mg/day to 8 mg/day) for more than 11 days or when taking the maximum dose of estrogen pills for up to 21 days. This was achieved by using twice-daily oral dydrogesterone tablets (20mg; Duphaston, Abbott, USA) and daily vaginal progesterone gel (90mg; Crinone, Merck Serono, Switzerland) until 10 weeks of gestation.

### Outcome measures

The serum β-hCG level was measured 14 days after embryo transfer. A value greater than 10 mIU/mL was considered positive. A transvaginal ultrasound scan was performed 4-5 weeks after embryo transfer to confirm clinical pregnancy by observing the gestational sac, regardless of its location. The clinical pregnancy rate (CPR) was defined by dividing the number of clinical pregnancies by the total number of transfer cycles, expressed as a percentage. Miscarriage was defined as a clinical intrauterine loss that occurred before 22 weeks of gestation. The miscarriage rate was calculated by dividing the number of miscarriages by the number of clinical pregnancies. Live birth was defined as an infant born alive after 28 weeks of gestation. The live birth rate (LBR) was calculated by dividing the number of live births by the total number of transfer cycles (18).

### Statistical analysis

Statistical analyses were conducted using SPSS software version 27.0. A significance level of P <0.05 was used. The normality of continuous parameters was evaluated using the Shapiro-Wilk test. All the continuous variables in this study were non-normally distributed and were reported as median (quartile 1-quartile 3) [M (P_25_, P_75_)] using nonparametric tests (Mann-Whitney U test). Categorical variables were presented as percentages (number/total number) and analyzed using the Chi-square test. A multivariate binary logistic regression analysis was performed to investigate the parameters associated with the live birth outcome.

## Results

A total of 336 cycles of PGT-SR that met the inclusion and exclusion criteria were included in the study (**Figure 1**). The pregnancy outcomes were as follows: positive β-hCG rate: 73.8%(248/336), CPR: 69.9%(228/336), miscarriage rate:14.0%(32/228), LBR: 57.4%(193/336). Additionally, two cycles resulted in ectopic pregnancies.

The cycles were categorized based on the live birth outcome. One group consisted of cycles that resulted in a live birth (n=193), while the other group consisted of cycles that did not result in a live birth result(n=143). The parameters of the study population and COS cycles are presented in **Table 1**. No significant differences were observed between the two groups in terms of the type of BCT (P=0.704), parental origin of BCT (P=0.523), maternal age (P=0.15), paternal age (P=0.716), anti-Mullerian hormone (AMH) levels (P=0.688), antral follicle count (AFC) (P=0.707), body mass index (BMI) (P=0.681), type of infertility (P=0.479), number of previous miscarriages (P=0.486), with PCOS (P=0.893), or with male factor (P=0.427). Meanwhile, in the COS cycles, there were no statistically significant differences in terms of the duration or total dose of gonadotropin (Gn) used (P=0.659 and P=0.914, respectively), estradiol levels (P=0.149), or endometrial thickness on trigger day (P=0.586). The number of oocytes retrieved (P=0.681), mature oocytes (P=0.416), fertilized oocytes (P=0.274), day 3 embryos (P=0.736), available blastocysts (P=0.204), biopsied blastocysts (P=0.316), and euploid blastocysts(P=0.131) were not significantly different between the two groups.

**Table 1:** Comparison of basic clinical data and parameters of controlled ovarian stimulation cycles between patients with and without a live birth.

Parameter	Live birth	No Live birth	P-value
No of patients	193	143	
Type of BCT (n)			0.704
reciprocal translocations	132	95	
Robertsonian translocations	61	48	
Parental origin of BCT (n)			0.523
female	85	68	
male	108	75	
Maternal age (years)	29(26-32)	29(27-32)	0.15
Paternal age (years)	30(27-33)	30(28-33)	0.716
AMH (ng/ml)	3.57(2.49-5.76)	4.05(2.44-5.59)	0.688
AFC	15(12-21)	15(11-22)	0.707
Female BMI (kg/m2)	23.10(20.87-25.40)	22.86(20.55-25.78)	0.681
Type of infertility			
Primary infertility	37.3% (72/193)	33.6% (48/143)	0.479
Secondary infertility	62.7% (121/193)	66.4% (95/143)	
No. of previous miscarriages	0(0-2)	1(0-2)	0.486
PCOS (n)	30	23	0.893
Male factor (n)*	39	24	0.427
Duration of Gn used(day)	10(9-12)	10(9-12)	0.659
Total dosage of Gn used (IU)	2100(1688-2700)	2100(1725-2700)	0.914
E2 level on trigger day (pg/ml)	2064(1292-3000)	2133(1538-3000)	0.149
Endometrial thickness on trigger day(mm)	10(8-12)	10(8-11)	0.586
No. of oocytes retrieved	13(10-18)	14(9-18)	0.681
MII	11(7-15)	12(7-15)	0.416
2PN	9(6-12)	9(6-14)	0.274
No. of Day 3 embryo available	8(5-11)	8(5-11)	0.736
No. of blastocyst available	5(3-7)	4(2-6)	0.204
No. of blastocyst biopsied	5(3-7)	4(2-6)	0.316
No. of Euploid embryos	2(1-3)	1(1-2)	0.131

BCT, balanced chromosomal translocations; AMH, anti-Mullerian hormone; AFC, antral follicle count; BMI, body mass index; PCOS, Polycystic Ovarian Syndrome; Gn, gonadotropin; hCG, human chorionic gonadotropin.

*: Sperm concentration <15 million/ml plus (progressive spermatozoa <32% or total motility <40%) plus morphology <4%, cryptozoospermia, or sperm extraction surgery.

Data are presented as median (quartile 1-quartile 3) or % (no./total no.).

The parameters of FBT cycles are shown in **Table 2**. The two groups had similar endometrial thickness at the start of progesterone treatment (P=0.167). There was no difference in the endometrial preparation protocol between the two groups (P=0.596). The live birth group had a higher percentage of blastocysts biopsied at day-5 compared to the group without a live birth (51.8% vs. 30.8%; P<0.001). Patients with a live birth had a significantly higher percentage of morphologically good quality blastocysts (≥BB) than those without a live birth (49.7% vs. 32.2%; P=0.001).

**Table 2:** Comparison of parameters of frozen-thawed embryo transfer cycles between patients with and without a live birth.

	Live birth	No Live birth	*P*-value
No. of patients	193	143	
Endometrial thickness (mm)	9.3 (8.5-10)	9(8.3-10)	0.167
Endometrial preparation protocols			0.596
natural	3.11% (6/193)	4.20% (6/143)	
HRT	96.89% (187/193)	95.80% (137/143)	
blastocyst developmental stage			
Day 5	51.8% (100/193)	30.8% (44/143)	<0.001
Day 6	48.2% (93/193)	69.2% (99/143)	
blastocyst quality			
good	49.7% (96/193)	32.2% (46/143)	0.001
poor	50.3% (97/193)	67.8% (97/143)	

HRT, hormone replacement therapy.

To identify factors that might affect the live birth outcome, multivariable binary logistic regression was used. The model included parameters with P<0.2, which were analyzed in univariable analyses between the two groups. These parameters consisted of maternal age, estradiol level on trigger day, number of euploid embryos, endometrial thickness at the start of progesterone treatment during the FBT cycles, and developmental stage and morphology of blastocysts. Additionally, the model included BMI and number of previous miscarriages, which were considered as significant factors in previous studies (19). **Table 3** indicates that blastocyst developmental stage and morphology significantly affected the probability of having a live birth. The likelihood of having a live birth increased when the blastocyst developmental stage changed from day 6 to day 5 (adjusted OR: 2.068, 95%CI 1.291-3.313; P=0.003). Meanwhile, transferring good-quality blastocysts increased the likelihood of having a live birth compared to transferring poor-quality blastocysts (adjusted OR: 1.697, 95%CI 1.039-2.773; P=0.035).

**Table 3:** Parameters related to live birth using binary logistic regression model.

Parameter	B	S.E.	Wald	P-value	adjusted odds ratio(aOR)	95%CI
Maternal age	-0.028	0.028	1.024	0.312	0.972	0.920-1.027
BMI	-0.012	0.032	0.147	0.702	0.988	0.929-1.051
No. of previous miscarriages	-0.03	0.103	0.086	0.77	0.97	0.793-1.187
E2 level on trigger day	0	0	1.658	0.198	1	1.0-1.0
No. of Euploid embryos	0.062	0.105	0.348	0.555	1.064	0.867-1.306
Endometrial thickness of FET cycles	0.082	0.077	1.146	0.284	1.085	0.934-1.261
blastocyst developmental stage						
Day 5	0.727	0.24	9.133	0.003	2.068	1.291-3.313
Day 6	reference					
blastocyst quality						
good	0.529	0.251	4.457	0.035	1.697	1.039-2.773
poor	reference					
constant	0.291	1.373	0.045	0.832	1.337	

## Discussion

The aim of this study was to investigate the parameters that may influence the live birth outcomes after the first single euploid FBT in patients with BCT. The results indicate that blastocyst morphology and developmental stage significantly affect the live birth outcome. Blastocysts available for biopsy at day 5 and have morphology≥BB have a higher likelihood of a live birth outcome. Maternal age, BMI, and previous miscarriages were similar between the two groups.

The LBR per single euploid FBT after PGT-SR cycles was found to be affected by the blastocyst developmental stage. However, this finding contradicted some previous studies. For instance, Wu et al. (20)reported no significant difference in the LBR between transfers of single day 5 and day 6 euploid good quality blastocysts (morphology≥4BB). Similarly, Liu et al. (21) found that the LBR of euploid blastocysts on day 5 was comparable to that of blastocysts on day 6 in young women (age < 35 years). Our findings are consistent with Li et al.’s study (9), which also found that day 5 euploid blastocysts had higher LBRs than day 6 blastocysts. Another study with a large sample size (day 5 blastocysts n=2321; day 6-7 blastocysts n=1497) reported similar results (22). Furthermore, in a recent meta-analysis reviewed 18 relative articles published between 2014 and 2021 and found that day 6-7 blastocysts (n=4627) had a significantly lower LBR per single euploid FBT than day 5 blastocysts (n=6716) (OR 0.56, 95%CI 0.48-0.63) (8). It is important to note that the euploid blastocysts analyzed in these studies were from PGT-A cycles, not PGT-SR cycles. The stage of the embryo development may reflect the metabolic health condition of the developing embryo (19). Delayed blastocyst formation may result from consequence of multiple minor embryonic functional defects that prevent an effective implantation and/or subsequent viable pregnancy.

Embryo morphology assessment is a valuable tool for embryo selection and is also suitable for selecting euploid blastocysts. However, it is still controversial whether the morphology of the blastocysts affects the LBR per single euploid FBT. Li et al. (9) found no significant association between the morphology of blastocysts and LBRs of euploid FBT. A multicenter retrospective study conducted by Capalbo et al. (23) found that the euploid embryos of different morphologies and developmental stage yielded a similar ongoing implantation rate. This result was consistent with the findings of the Viñals Gonzalez’s study in advanced maternal age (24). However, Cimadomo et al. (25) found that poor-quality blastocysts had a worse prognosis compared to good-quality blastocysts in advanced maternal age. In addition, a meta-analysis revealed that ICM and TE with a score of C, whether considered together or separately, were linked to a decreased LBR per euploid FBT (8). Regarding overall blastocyst quality, blastocysts of poor quality (n=722) had a significantly lower LBR per single euploid FBT compared to blastocysts of good quality (n=4384) (OR 0.20, 95%CI 0.24-0.67). The euploid blastocysts analyzed in these studies were from PGT-A cycles. Due to the subjective nature of morphological grading of embryos, there is a lack of reproducibility between different IVF laboratories (26). This may be one of the reasons for the inconsistent results. Improving inter- and intra-observer concordance would be helpful. In the future, time-lapse technology combined with artificial intelligence (AI) may offer promising prospects (27).

It is well known that increasing maternal age (especially ≥ 35 years) is associated with decreased reproductive capacity due to decreased ovarian reserve and increased aneuploidy. Additionally, Qiao et al. (28) suggested that the age-related reproductive decline was not only due to increasing aneuploid rates but also to metabolic and epigenetic changes in the embryos. The effect of age on endometrial function is also a concern (29). A meta-analysis conducted recently found that increasing maternal age was linked to a decrease in LBR after euploid FBT following PGT-A (30). However, Idowu et al. (12) and Ogur et al. (31) found no significant difference in the LBR between mothers aged ≥ 35 years and those aged < 35 years (n=18 vs 36, and n=49 vs 164, respectively) after euploid FBT in couples with structural chromosomal rearrangements after PGT-SR. Mateu-Brull et al. (32) found that clinical outcome according to the age group (< 38 years vs 38-42 years) showed no statistical differences for each type of BCT. The present study also found that maternal age had no significant effect on the LBR, which was presented as a continuous variable. Of the 336 patients, 41 were 35 years or older, representing 12.2% of the total. The variations in the results can be attributed to the different chromosomal conditions of the study populations. On the other hand, carriers of BCT tend to be younger. The maternal age of the study population was 29 (26–32) years.

The impact of increased maternal BMI on the LBR after euploid FBT remains uncertain. Boynukalin et al. (19) and Li et al. (9) reported a significant association between BMI and LBR after euploid FBT. Cozzolino et al. (33) and Meng et al. (34) found that obese women (BMI ≥ 30 kg/m^2^) had a significantly lower LBR after euploid FBT compared to non-obese women. Another study showed that the LBR after euploid FBT was significantly reduced in overweight women (BMI ≥ 25 kg/m^2^), especially in obese women (BMI ≥ 30 kg/m^2^) (35). However, some studies have not found a significant association between BMI and LBR after euploid FBT. For instance, In Zhou et al.’s study (36), BMI was comparable among live birth, miscarriage and not-implanted groups. Similarly, in Kim et al.’s study (37), the LBR was similar across all weight categories, including underweight, normal, overweight, and obese groups. And the euploid blastocysts in these studies were from PGT-A cycles. Increased BMI reflects maternal nutritional imbalances and may also be the result of endocrine dysfunction and immunologic factors, and may impair uterine receptivity, alter folliculogenesis, and compromise oocyte quality (38), which could be the cause of RM and RIF. Therefore, there may be confounding factors to consider. The present study found no significant association between BMI and LBR after euploid FBT in BCT carriers. The population in this study had a lower BMI of 22.97(20.70-25.60), which may partially explain the lack of association. Of the 336 patients, 77(21.2%) patients were overweight, with LBR (51.9%) and 21 (5.8%) patients were obese, with LBR (66.7%). Most of the previous studies reporting lower pregnancy rates in obese patients were based on fresh embryo transfers (39, 40). The studies by Prost et al. (41) and Insogna et al. (42) which focused on FBT cycles, found that female obesity did not affect LBR. With improved embryo-endometrial synchrony, frozen-thawed embryo transfer may be beneficial for overweight and obese patients. In addition, corpus luteum function may be impaired in overweight or obese patients (35), and increased progestin supplementation may reduce the high risk of miscarriage (37). This finding does not negate the fact that overweight and obesity have adverse effects on maternal and fetal health, such as pregnancy-induced hypertension, gestational diabetes, and fetal growth restriction, so weight intervention is recommended prior to euploid FBT.

Women who have experienced a miscarriage in the past are at a higher risk of experiencing a subsequent miscarriage if they conceive naturally (43). It is uncertain whether previous miscarriages influence LBR after euploid FBT. LBR decreased as the number of miscarriages increased, according to Boynukalin et al (19). Liu et al’s study (44) showed that the group with idiopathic recurrent pregnancy loss (iPRL) had a significantly lower LBR compared to the control group. However, Cimadomo et al. (45) found no difference in LBR after euploid FBT (n=1580) among patients with 0, 1, or more than 1 previous miscarriages after PGT-A cycles, which was consistent with the study of Wang et al. (46). Ni et al. (47) investigated the association between previous pregnancy failures and pregnancy outcomes after PGT-A. They found that a history of ≥4 early miscarriages was significantly associated with a higher early miscarriage rate, but was not directly associated with lower LBR after logistic regression. They did not find a clear association between a history of 2 previous early miscarriages and pregnancy outcomes. In the present study, the number of previous miscarriages did not affect LBR after euploid FBT, which is a reassuring. This means that a history of recurrent pregnancy loss has no prognostic value in predicting live birth outcome in euploid FBT after PGT-SR. However, it should be noted that the sample size of high-order miscarriages in our study was relatively limited. Among the 336 patients, 27 (8.0%) patients had 3 previous miscarriages, with LBR (59.3%), and 10 (3.0%) patients had ≥4 previous miscarriages, with LBR (50.0%). Another study, not focused on the PGT cycles, reported by Qiu et al. (48) found that the history of recurrent pregnancy loss was not significantly associated with miscarriage and LBR of the first *in vitro* fertilization/ICSI frozen embryo transfer or intrauterine insemination cycles, suggesting that fertility treatment itself may mitigate the effect of miscarriage history on subsequent pregnancy. These findings are encouraging.

Whether the parental origin of BCT affects the outcomes of PGT-SR is an interesting question. Tong et al. (49) found that the parental origin of BCT had no significant difference on the laboratory results and ploidy results. In addition, Insogna et al. (50) also found that the type and parental origin of BCT had no significant difference on pregnancy outcomes. Mayeur et al. (51) reported that LBR were not significantly different between female and male BCT carriers. Mateu-Brull et al. (32) observed that ongoing CPR per transfer was comparable between BCT types, and male reciprocal translocation carriers had higher CPR per transfer after day 3 biopsy, but there was no sex effect for CPR per transfer after day 5/6 biopsy. The present study did not find an association between the parental origin of the BCT and the LBR of single euploid FBT. Overall, this means that clinical decisions can be made without regardless of the type and parental origin of the BCT.

## Strengths and limitations

This study investigated the factors affecting the LBR after euploid FBT in the population of BCT carriers who are at high risk of adverse pregnancy outcomes undergoing PGT-SR. Previous studies have mainly focused on the patients with AMA, RIF, or RM undergoing PGT-A. The present study focused only on the population of BCT carriers with euploid blastocysts after PGT-SR. It does not represent the entire population of BCT carriers, who face several uncontrollable factors throughout the PGT-SR process, such as the chromosome involved in the structural rearrangement, ovarian reserve, ovarian response, and embryo development before obtaining euploid blastocysts. After euploid blastocysts are obtained, patients may experience anxiety while waiting for the outcome of FBT. This study provides additional information to help alleviate their anxiety and set appropriate expectations. We only included the first single euploid FBT cycles to eliminate confounding factors. However, it has some limitations. This study solely focused on the live birth outcomes, and did not evaluate other clinical outcomes, such as implantation rate and miscarriage rate. Additionally, the retrospective study design carries a certain risk of bias. Furthermore, the sample size is not large enough and has limited power. Lastly, this is a single-center study, and the conclusions can only reflect the results of the center’s clinical and laboratory practices. Large-scale multicenter studies will be necessary to confirm our current findings in the future.

In conclusion, blastocyst morphology and developmental stage are the factors that influence the live birth outcome after single euploid FBT in BCT carriers. This information is valuable for clinical counseling and for electing the embryo to transfer.

## Data availability statement

The raw data supporting the conclusions of this article will be made available by the authors, without undue reservation.

## Ethics statement

The studies involving humans were approved by the Ethics Committee of Henan Provincial People's Hospital. Written informed consent to participate in this study was not required from the participants or the participants’ legal guardians/next of kin in accordance with the national legislation and the institutional requirements.

## Author contributions

ZR: Data curation, Formal analysis, Writing – original draft. HY: Data curation, Formal analysis, Writing – review & editing. CC: Writing – review & editing, Data curation, Formal analysis. ZC: Funding acquisition, Supervision, Writing – review & editing.

## References

[1] Nielsen J Wohlert M. Chromosome abnormalities found among 34,910 newborn children: results from a 13-year incidence study in Arhus, Denmark. Hum Genet. (1991) 87:81–3. doi: 10.1007/bf01213097 2037286

[2] Fiorentino F Spizzichino L Bono S Biricik A Kokkali G Rienzi L. Pgd for reciprocal and Robertsonian translocations using array comparative genomic hybridization. Hum Reprod (Oxford England). (2011) 26:1925–35. doi: 10.1093/humrep/der082 21489979

[3] Carvalho F Coonen E Goossens V Kokkali G Rubio C Meijer-Hoogeveen M. Eshre pgt consortium good practice recommendations for the organisation of pgt. Hum Reprod Open. (2020) 2020:hoaa021. doi: 10.1093/hropen/hoaa021 32524036 PMC7257038

[4] Iews M Tan J Taskin O Alfaraj S AbdelHafez FF Abdellah AH. Does preimplantation genetic diagnosis improve reproductive outcome in couples with recurrent pregnancy loss owing to structural chromosomal rearrangement? A systematic review. Reprod Biomed Online. (2018) 36:677–85. doi: 10.1016/j.rbmo.2018.03.005 29627226

[5] Li S Zheng PS Ma HM Feng Q Zhang YR Li QS. Systematic review of subsequent pregnancy outcomes in couples with parental abnormal chromosomal karyotypes and recurrent pregnancy loss. Fertil Steril. (2022) 118:906–14. doi: 10.1016/j.fertnstert.2022.08.008 36175209

[6] Dahdouh EM Balayla J García-Velasco JA. Comprehensive chromosome screening improves embryo selection: A meta-analysis. Fertil Steril. (2015) 104:1503–12. doi: 10.1016/j.fertnstert.2015.08.038 26385405

[7] Chen M Wei S Hu J Quan S. Can comprehensive chromosome screening technology improve ivf/icsi outcomes? A meta-analysis. PloS One. (2015) 10:e0140779. doi: 10.1371/journal.pone.0140779 26470028 PMC4607161

[8] Cimadomo D Rienzi L Conforti A Forman E Canosa S Innocenti F. Opening the black box: why do euploid blastocysts fail to implant? A systematic review and meta-analysis. Hum Reprod Update. (2023) 29:570–633. doi: 10.1093/humupd/dmad010 37192834

[9] Li N Guan Y Ren B Zhang Y Du Y Kong H. Effect of blastocyst morphology and developmental rate on euploidy and live birth rates in preimplantation genetic testing for aneuploidy cycles with single-embryo transfer. Front Endocrinol. (2022) 13:858042. doi: 10.3389/fendo.2022.858042 PMC9044033 35498424

[10] Bender Atik R Christiansen OB Elson J Kolte AM Lewis S Middeldorp S. Eshre guideline: recurrent pregnancy loss. Hum Reprod Open. (2018) 2018:hoy004. doi: 10.1093/hropen/hoy004 31486805 PMC6276652

[11] Cimadomo D de Los Santos MJ Griesinger G Lainas G Le Clef N McLernon DJ. Eshre good practice recommendations on recurrent implantation failure. Hum Reprod Open. (2023) 2023:hoad023. doi: 10.1093/hropen/hoad023 37332387 PMC10270320

[12] Idowu D Merrion K Wemmer N Mash JG Pettersen B Kijacic D. Pregnancy outcomes following 24-chromosome preimplantation genetic diagnosis in couples with balanced reciprocal or Robertsonian translocations. Fertil Steril. (2015) 103:1037–42. doi: 10.1016/j.fertnstert.2014.12.118 25712573

[13] Harper JC Wilton L Traeger-Synodinos J Goossens V Moutou C SenGupta SB. The eshre pgd consortium: 10 years of data collection. Hum Reprod Update. (2012) 18:234–47. doi: 10.1093/humupd/dmr052 22343781

[14] Boynukalin FK Gultomruk M Turgut NE Rubio C Rodrigo L Yarkiner Z. The impact of patient, embryo, and translocation characteristics on the ploidy status of young couples undergoing preimplantation genetic testing for structural rearrangements (Pgt-sr) by next generation sequencing (Ngs). J Assisted Reprod Genet. (2021) 38:387–96. doi: 10.1007/s10815-020-02054-4 PMC7884505 33398513

[15] Chen Y Guo J Zhang Q Zhang C. Insulin resistance is a risk factor for early miscarriage and macrosomia in patients with polycystic ovary syndrome from the first embryo transfer cycle: A retrospective cohort study. Front Endocrinol. (2022) 13:853473. doi: 10.3389/fendo.2022.853473 PMC9046670 35498421

[16] Zhang S Yin Y Li Q Zhang C. Comparison of cumulative live birth rates between gnrh-a and ppos in low-prognosis patients according to Poseidon criteria: A cohort study. Front Endocrinol. (2021) 12:644456. doi: 10.3389/fendo.2021.644456 PMC8256850 34234739

[17] Gardner DK Surrey E Minjarez D Leitz A Stevens J Schoolcraft WB. Single blastocyst transfer: A prospective randomized trial. Fertil Steril. (2004) 81:551–5. doi: 10.1016/j.fertnstert.2003.07.023 15037401

[18] Zegers-Hochschild F Adamson GD Dyer S Racowsky C de Mouzon J Sokol R. The international glossary on infertility and fertility care, 2017. Hum Reprod (Oxford England). (2017) 32:1786–801. doi: 10.1093/humrep/dex234 PMC5850297 29117321

[19] Boynukalin FK Gultomruk M Cavkaytar S Turgut E Findikli N Serdarogullari M. Parameters impacting the live birth rate per transfer after frozen single euploid blastocyst transfer. PloS One. (2020) 15:e0227619. doi: 10.1371/journal.pone.0227619 31929583 PMC6957140

[20] Wu TF Chen MJ Lee MS Huang CC Ho ST Cheng EH. Comparison of clinical outcome between day 5 and day 6 single blastocyst transfers in cycles undergoing preimplantation genetic testing for aneuploidy. Taiwan J Obstet Gynecol. (2023) 62:429–33. doi: 10.1016/j.tjog.2023.03.005 37188448

[21] Liu Y Zhang X Xu Y Li R Cai B Ding C. Similar implantation competence in euploid blastocysts developed on day 5 or day 6 in young women: A retrospective cohort study. Hum Fertil (Cambridge England). (2023) 26:918–26. doi: 10.1080/14647273.2021.2021454 34983269

[22] Hernandez-Nieto C Lee JA Slifkin R Sandler B Copperman AB Flisser E. What is the reproductive potential of day 7 euploid embryos? Hum Reprod (Oxford England). (2019) 34:1697–706. doi: 10.1093/humrep/dez129 31398251

[23] Capalbo A Rienzi L Cimadomo D Maggiulli R Elliott T Wright G. Correlation between standard blastocyst morphology, euploidy and implantation: an observational study in two centers involving 956 screened blastocysts. Hum Reprod (Oxford England). (2014) 29:1173–81. doi: 10.1093/humrep/deu033 24578475

[24] Viñals Gonzalez X Odia R Naja R Serhal P Saab W Seshadri S. Euploid blastocysts implant irrespective of their morphology after ngs-(Pgt-a) testing in advanced maternal age patients. J Assisted Reprod Genet. (2019) 36:1623–9. doi: 10.1007/s10815-019-01496-9 PMC6707991 31165389

[25] Cimadomo D Soscia D Vaiarelli A Maggiulli R Capalbo A Ubaldi FM. Looking past the appearance: A comprehensive description of the clinical contribution of poor-quality blastocysts to increase live birth rates during cycles with aneuploidy testing. Hum Reprod (Oxford England). (2019) 34:1206–14. doi: 10.1093/humrep/dez078 31247100

[26] Cimadomo D Sosa Fernandez L Soscia D Fabozzi G Benini F Cesana A. Inter-centre reliability in embryo grading across several ivf clinics is limited: implications for embryo selection. Reprod Biomed Online. (2022) 44:39–48. doi: 10.1016/j.rbmo.2021.09.022 34819249

[27] Apter S Ebner T Freour T Guns Y Kovacic B Le Clef N. Good practice recommendations for the use of time-lapse technology(†). Hum Reprod Open. (2020) 2020:hoaa008. doi: 10.1093/hropen/hoaa008 32206731 PMC7081060

[28] Qiao J Wang ZB Feng HL Miao YL Wang Q Yu Y. The root of reduced fertility in aged women and possible therapentic options: current status and future perspects. Mol Aspects Med. (2014) 38:54–85. doi: 10.1016/j.mam.2013.06.001 23796757

[29] Pathare ADS Loid M Saare M Gidlöf SB Zamani Esteki M Acharya G. Endometrial receptivity in women of advanced age: an underrated factor in infertility. Hum Reprod Update. (2023) 29:773–93. doi: 10.1093/humupd/dmad019 PMC10628506 37468438

[30] Vitagliano A Paffoni A Viganò P. Does Maternal Age Affect Assisted Reproduction Technology Success Rates after Euploid Embryo Transfer? A systematic Review and Meta-Analysis. Fertil Steril. (2023) 120:251–65. doi: 10.1016/j.fertnstert.2023.02.036 36878347

[31] Ogur C Kahraman S Griffin DK Cinar Yapan C Tufekci MA Cetinkaya M. Pgt for structural chromosomal rearrangements in 300 couples reveals specific risk factors but an interchromosomal effect is unlikely. Reprod Biomed Online. (2023) 46:713–27. doi: 10.1016/j.rbmo.2022.07.016 36803887

[32] Mateu-Brull E Rodrigo L Peinado V Mercader A Campos-Galindo I Bronet F. Interchromosomal effect in carriers of translocations and inversions assessed by preimplantation genetic testing for structural rearrangements (Pgt-sr). J Assisted Reprod Genet. (2019) 36:2547–55. doi: 10.1007/s10815-019-01593-9 PMC6911137 31696386

[33] Cozzolino M García-Velasco JA Meseguer M Pellicer A Bellver J. Female obesity increases the risk of miscarriage of euploid embryos. Fertil Steril. (2021) 115:1495–502. doi: 10.1016/j.fertnstert.2020.09.139 33267960

[34] Meng F Goldsammler M Wantman E Buyuk E Jindal SK. Live birth rate from euploid blastocysts is not associated with infertility etiology or oocyte source following frozen-thawed embryo transfer (Fet): analysis of 4148 cycles reported to Sart Cors. J Assisted Reprod Genet. (2021) 38:185–92. doi: 10.1007/s10815-020-01996-z PMC7822968 33155088

[35] Tremellen K Pearce K Zander-Fox D. Increased miscarriage of euploid pregnancies in obese women undergoing cryopreserved embryo transfer. Reprod Biomed Online. (2017) 34:90–7. doi: 10.1016/j.rbmo.2016.09.011 27789185

[36] Zhou X Liu X Shi W Ye M Chen S Xu C. Mitochondrial DNA content may not be a reliable screening biomarker for live birth after single euploid blastocyst transfer. Front Endocrinol. (2021) 12:762976. doi: 10.3389/fendo.2021.762976 PMC8637898 34867804

[37] Kim J Patounakis G Juneau C Morin S Neal S Bergh P. The appraisal of body content (Abc) trial: increased male or female adiposity does not significantly impact *in vitro* fertilization laboratory or clinical outcomes. Fertil Steril. (2021) 116:444–52. doi: 10.1016/j.fertnstert.2020.12.037 33581854

[38] Broughton DE Moley KH. Obesity and female infertility: potential mediators of obesity's impact. Fertil Steril. (2017) 107:840–7. doi: 10.1016/j.fertnstert.2017.01.017 28292619

[39] Luke B Brown MB Stern JE Missmer SA Fujimoto VY Leach R. Female obesity adversely affects assisted reproductive technology (Art) pregnancy and live birth rates. Hum Reprod (Oxford England). (2011) 26:245–52. doi: 10.1093/humrep/deq306 21071489

[40] Provost MP Acharya KS Acharya CR Yeh JS Steward RG Eaton JL. Pregnancy outcomes decline with increasing body mass index: analysis of 239,127 fresh autologous in vitro fertilization cycles from the 2008-2010 society for assisted reproductive technology registry. Fertil Steril. (2016) 105:663–9. doi: 10.1016/j.fertnstert.2015.11.008 26627120

[41] Prost E Reignier A Leperlier F Caillet P Barrière P Fréour T. Female obesity does not impact live birth rate after frozen-thawed blastocyst transfer. Hum Reprod (Oxford England). (2020) 35:859–65. doi: 10.1093/humrep/deaa010 32170315

[42] Insogna IG Lee MS Reimers RM Toth TL. Neutral effect of body mass index on implantation rate after frozen-thawed blastocyst transfer. Fertil Steril. (2017) 108:770–6.e1. doi: 10.1016/j.fertnstert.2017.08.024 28985909

[43] Magnus MC Wilcox AJ Morken NH Weinberg CR Håberg SE. Role of maternal age and pregnancy history in risk of miscarriage: prospective register based study. BMJ (Clinical Res ed). (2019) 364:l869. doi: 10.1136/bmj.l869 PMC6425455 30894356

[44] Liu XY Fan Q Wang J Li R Xu Y Guo J. Higher chromosomal abnormality rate in blastocysts from young patients with idiopathic recurrent pregnancy loss. Fertil Steril. (2020) 113:853–64. doi: 10.1016/j.fertnstert.2019.11.016 32228881

[45] Cimadomo D Capalbo A Dovere L Tacconi L Soscia D Giancani A. Leave the past behind: women's reproductive history shows no association with blastocysts' Euploidy and limited association with live birth rates after euploid embryo transfers. Hum Reprod (Oxford England). (2021) 36:929–40. doi: 10.1093/humrep/deab014 33608730

[46] Wang A Kort J Westphal L. Miscarriage history association with euploid embryo transfer outcomes. Reprod Biomed Online. (2019) 39:617–23. doi: 10.1016/j.rbmo.2019.05.011 31395518

[47] Ni T Wu Q Zhu Y Jiang W Zhang Q Li Y. Comprehensive analysis of the associations between previous pregnancy failures and blastocyst aneuploidy as well as pregnancy outcomes after pgt-A. J Assisted Reprod Genet. (2020) 37:579–88. doi: 10.1007/s10815-020-01722-9 PMC7125264 32103397

[48] Qiu J Du T Li W Zhao M Zhao D Wang Y. Impact of recurrent pregnancy loss history on reproductive outcomes in women undergoing fertility treatment. Am J Obstetrics Gynecol. (2023) 228:66.e1–.e9. doi: 10.1016/j.ajog.2022.08.014 35970200

[49] Tong J Niu Y Wan A Zhang T. Effect of parental origin and predictors for obtaining a euploid embryo in balanced translocation carriers. Reprod Biomed Online. (2022) 44:72–9. doi: 10.1016/j.rbmo.2021.09.007 34865999

[50] Insogna IG Lanes A Dobson L Ginsburg ES Racowsky C Yanushpolsky E. Blastocyst conversion rate and ploidy in patients with structural rearrangements. J Assisted Reprod Genet. (2021) 38:1143–51. doi: 10.1007/s10815-021-02131-2 PMC8190241 33656620

[51] Mayeur A Ahdad N Hesters L Grynberg M Romana S Sonigo C. Does the prognosis after pgt for structural rearrangement differ between female and male translocation carriers? Reprod Biomed Online. (2020) 40:684–92. doi: 10.1016/j.rbmo.2020.01.025 32334941

